# A new generative adversarial network for medical images super resolution

**DOI:** 10.1038/s41598-022-13658-4

**Published:** 2022-06-09

**Authors:** Waqar Ahmad, Hazrat Ali, Zubair Shah, Shoaib Azmat

**Affiliations:** 1 Department of Electrical and Computer Engineering, COMSATS University Islamabad, Abbottabad Campus, Abbottabad, Pakistan; 2 National Center of Artificial Intelligence, Peshawar, Pakistan; 3 College of Science and Engineering, Hamad Bin Khalifa University, Doha, Qatar

**Keywords:** Engineering, Mathematics and computing, Medical research

## Abstract

For medical image analysis, there is always an immense need for rich details in an image. Typically, the diagnosis will be served best if the fine details in the image are retained and the image is available in high resolution. In medical imaging, acquiring high-resolution images is challenging and costly as it requires sophisticated and expensive instruments, trained human resources, and often causes operation delays. Deep learning based super resolution techniques can help us to extract rich details from a low-resolution image acquired using the existing devices. In this paper, we propose a new Generative Adversarial Network (GAN) based architecture for medical images, which maps low-resolution medical images to high-resolution images. The proposed architecture is divided into three steps. In the first step, we use a multi-path architecture to extract shallow features on multiple scales instead of single scale. In the second step, we use a ResNet34 architecture to extract deep features and upscale the features map by a factor of two. In the third step, we extract features of the upscaled version of the image using a residual connection-based mini-CNN and again upscale the feature map by a factor of two. The progressive upscaling overcomes the limitation for previous methods in generating true colors. Finally, we use a reconstruction convolutional layer to map back the upscaled features to a high-resolution image. Our addition of an extra loss term helps in overcoming large errors, thus, generating more realistic and smooth images. We evaluate the proposed architecture on four different medical image modalities: (1) the DRIVE and STARE datasets of retinal fundoscopy images, (2) the BraTS dataset of brain MRI, (3) the ISIC skin cancer dataset of dermoscopy images, and (4) the CAMUS dataset of cardiac ultrasound images. The proposed architecture achieves superior accuracy compared to other state-of-the-art super-resolution architectures.

## Introduction

High-resolution (HR) images contain detailed information structures as compared to low-resolution (LR) images. Usually, expensive image acquisition devices are used to acquire HR images. This results in a long acquisition time and a relatively low signal-to-noise ratio^1^. Instead of using such a costly method for image acquisition, we can retrieve HR images from LR images using super-resolution (SR) methods. Technically, image super-resolution is a technique to reconstruct the high-frequency contents in an LR image. Using super-resolution methods, we only need LR images, which in turn reduce the image acquisition complexities. However, super-resolution is a challenging task. In the last few years, super-resolution methods have been proposed for both natural images and medical images. In the case of medical images, fine details such as small anatomical structures carry important information that is useful for diagnostic purposes. For example, in brain MRI, the small structure details around a tumor help in diagnosing the growth rate and the origin of the tumor^2,3^. Similarly, in retinal images, the correct identification of fine vessels helps diagnose the swelling of vessels, which is a symptom of hypertensive retinopathy. Therefore, it is not desirable to allow the artifacts introduced by super-resolution methods as these may have an adversarial effect on the diagnosis. Over the years, the super-resolution methods proposed for both natural images and medical images can broadly be categorized into two categories: traditional methods and deep learning methods. We discuss both the categories below.

### Traditional machine learning methods

The methods used for image super-resolution in this category are mainly divided into three types, i.e., interpolation-based methods, reconstruction-based methods, and learning-based methods. The early and primary methods include interpolation-based methods such as linear interpolation and bicubic interpolation. Zhang et al.^4^ used an interpolation approach for image super-resolution. The authors transformed the LR image to discrete cosine transform (DCT) domain and applied interpolation methods to estimate high frequencies. The interpolation-based methods for upscaling by a significant factor such as 6x or 8x typically result in blurred output images.

The reconstruction-based methods perform better than interpolation-based methods. Sun et al.^5^ used the gradient profile method to map LR images to HR images. The authors used a non-local mean filter to extract gradient profile information, which was used to map the LR images to HR images. Based on the same idea, Zhang et al.^6^ also used gradient knowledge to reconstruct HR images. In^7^, the authors proposed a reconstruction-based method in which non-local mean features and handcrafted features such as Gabor wavelet and sparse domain selection-based features were used for HR image reconstruction. Protter et al.^8^ used the gradient profile method for the reconstruction of a sequence of images such as videos. These methods are also applied to medical images as reported in^9,10^. By using the gradient information, these methods outperformed interpolation methods to map LR images to HR images. The limitation of these methods is that one must design and use robust filters to extract gradient information. If the LR images have no clear gradient, then the performance of the SR method will be heavily affected.

The learning-based methods have recently attracted more attention for image super-resolution. For example, Freedman et al.^11^ and Yang et al.^12^ extracted highly localized patches from the input LR image and defined a mapping function to map these LR patches to an HR image. In^13^, the authors used a random forest classifier to classify the image space into multiple subspaces and then used a regression model to map the extracted patches to HR patches. The above-discussed methods are some of the traditional machine learning-based SR methods.

### Deep learning-based methods

Recently, deep learning-based approaches have been explored for image super-resolution. These approaches have outperformed many traditional methods. In^14^, a feed-forward convolutional neural network (SRCNN) was used for feature extraction from LR images. The features were then up-sampled using the bilinear interpolation method. The architecture works in an end-to-end manner. SRCNN reported better results compared to the traditional methods. Kim et al.^15^ used deep recursive CNN for image super-resolution to extract complex features. The method is divided into three parts: the embedding network used for basic features extraction, the inference network used for deep feature extraction, and the reconstruction layer that maps back the features to a HR image. The deep recursive approach is also used in^16^ and^17^. With such deep networks, the vanishing gradient problem is a major concern. A dense skip connection was used by^18^ to overcome the problem of vanishing gradient.

For up-sampling in CNNs, the sub-pixel convolutional layer was introduced in^19^, and an enhanced sub-pixel convolutional network was proposed in^20^. The sub-pixel up-sampling layer outperformed both interpolation-based up-sampling and transpose convolution-based upsampling methods. Zhang et al.^21^ proposed a channel attention mechanism to improve the performance of image SR methods. The authors used local receptive fields to incorporate channel-dependent features. The limitation of the method is that it might miss some non-local features. Dai et al.^22^ used both local and non-local receptive fields for feature extraction to handle this problem. The performance of the super-resolution method relies on the robustness of the extracted features. Due to better performance, the ResNet34^23^ architecture was adopted by authors in^24,25,25^. Using the same idea, Ahn et al.^26^ used a cascade of the residual network blocks.

To extract robust features, Lim et al, in^27^ used ResNet-based architecture for feature extraction. In addition, Lim et al.^27^ modified the traditional ResNet architecture by removing the batch normalization layer after each convolutional layer and proposed enhanced deep super-resolution architecture (EDSR). The limitation of the above methods is that all these methods only focus on the high peak-signal-to-noise ratio (PSNR). To obtain high PSNR, the authors only used mean square error (MSE) or mean absolute error (MAE) losses; however, the two losses make the resultant image more smooth and do not preserve the fine contents of the image. Pourya et al.^28^ has proposed a progressive dilated convolution neural network for SR. The proposed method has shown superior performance compared to SRCNN and other existing models on natural images while utilizing less computational resources.

Among deep learning-based super-resolution methods, Generative Adversarial Networks (GAN) based methods have demonstrated significant improvement^29,30^. To overcome the limitations of CNN-based SR methods, the GAN architecture uses the perceptual or content based loss function, i.e., the VGG19 loss. The first GAN-based SR method, called SRGAN, was proposed in^31^. The generator of the SRGAN is a ResNet34 architecture that extracts features from LR images. For up-sampling, a sub-pixel convolution layer was used. This method was further modified by Wang et al.^32^. They used multi-residual networks, i.e., residual-in-residual dense blocks. In addition, the authors eliminated the batch normalization layer after every convolutional layer. The elimination of the batch normalization layer improved the performance of traditional SRGAN architecture. Using multiple residual blocks or nested residual blocks increases computational complexity. Jiang et al.^33^ proposed edge-enhanced GAN for super-resolution of satellite images. The architecture comprises two networks: ultra-dense subnetwork (UDSN), which extracts features and obtains high resolution features having sharp edges, and edge-enhanced sub-network (EESN), which enhances the extracted sharp edges and removes artifacts produced during the UDSN feature extraction process. In^34^, the authors used a GAN and a saliency map to generate retinal fundus HR images. The effect of saliency map is used to minimize the GAN cost function, which effectively improves the performance of the generation of HR images using GAN. The limitation of this method is that, to improve the neural network’s performance, saliency maps are extracted separately, and efficient filters should be used to obtain robust saliency maps. Chen et al.^35^ proposed a multilevel densely connected SR network (mDCSRN) for brain structural MRI images super-resolution. The authors reported good accuracy with the proposed network and reported a sixfold increase in speed compared to other GAN-based models. MedSRGAN is proposed in^36^. The authors modified the RCAN^21^ architecture by replacing the global pooling with a 1x1 convolution layer. Multiple residual-in-residual (RIR) blocks are used for feature extraction and sub-pixel convolutional layer for 4x upscaling. The discriminator network in this work takes pair of images, i.e. (LR, HR) images pair and (LR, SR) images pair, to discriminate between a generated HR image and an original HR image. The limitation of the above-mentioned GAN-based methods is that they use very deep networks on a single scale, i.e., filter size three, which may miss some of the large-scale features. Furthermore, these methods perform the up-sampling in a single step rather than up-sampling progressively.

In this paper, we propose a GAN-based image super-resolution method for medical images. In the proposed method, we use SRGAN^31^ as a baseline and introduce significant modifications to its generator architecture. In^31^, the authors used ResNet34 architecture in the generator to extract single-scale features from LR images and then used a sub-pixel convolutional layer for 4x upscaling. In our work, we modify the structure of the generator network to extract shallow features on different scales. We evaluate our model on publicly available medical imaging datasets, i.e., retinal images datasets (STARE and DRIVE)^37,38^, ISIC skin lesion segmentation dataset^39^, BraTS 2018 dataset of brain MRI^40^, and the CAMUS dataset of cardiac ultrasound images^41^. The main contributions of this work are:

- We present a new GAN-based super-resolution model for medical images. The model extracts shallow features on different scales, i.e., filter sizes 3, 5, and 7. In other GAN-based networks^31,36^, the authors extracted only single-scale features, i.e., filter size 3.
- Unlike other GAN-based models, our model splits the process of upscaling into two steps. In the first step, shallow features are extracted, followed by deep features extraction using ResNet34 architecture, and then 2x upscaling is performed. In the second step, more complex features are calculated from the 2x upscaled version, and then 2x upscaling is performed again. This progressive upscaling helps in preserving fine details at each scale.
- In our model, we add one more loss function, i.e., mean absolute error (L1 loss), to the loss functions of SRGAN^31^, which in turn results in performance improvement.
- The super-resolution performance for our proposed method using multi-scaled features, progressive up-sampling with added loss function, outperforms the current state-of-the-art SR methods on multiple medical imaging modalities.

## Methods

Our architecture is a multi-path and progressive upscaling GAN. In the following text, we present the generator architecture, the discriminator architecture and the loss functions.

### Generator network

**Figure 1:**
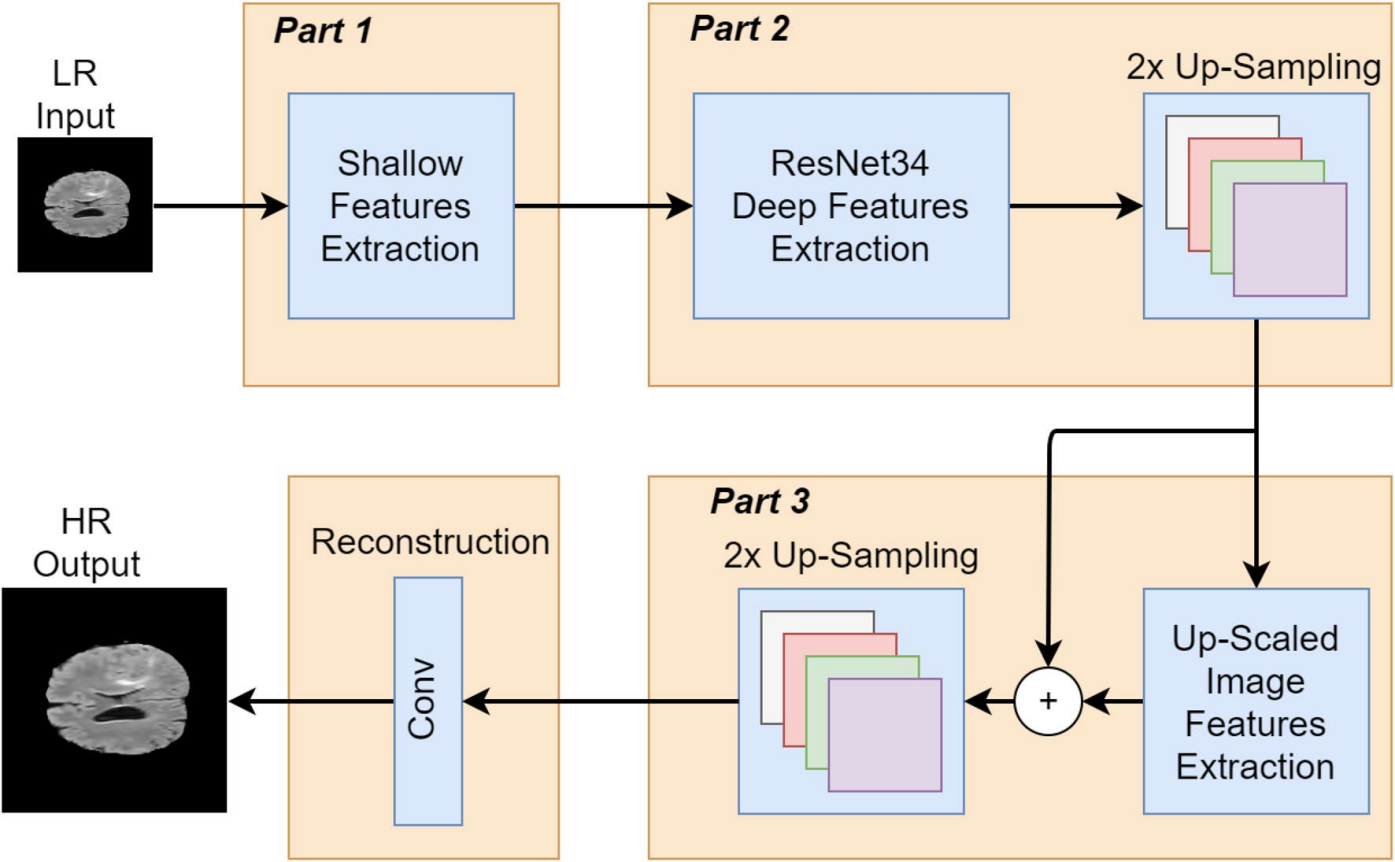
An overall block diagram to show the two rounds of the features extraction and 2x upscaling.

To overcome the shortcomings and improve the accuracy of the SRGAN^31^ for medical images, we propose a novel generator network with the following three major changes. The block diagram of the proposed architecture illustrating the changes, is shown in Fig. 1. The major components are:

- Shallow features extraction.
- Deep features extraction and 2x upscaling.
- Features extraction of upscaled version and 2x upscaling.

#### Shallow features extraction

**Figure 2:**
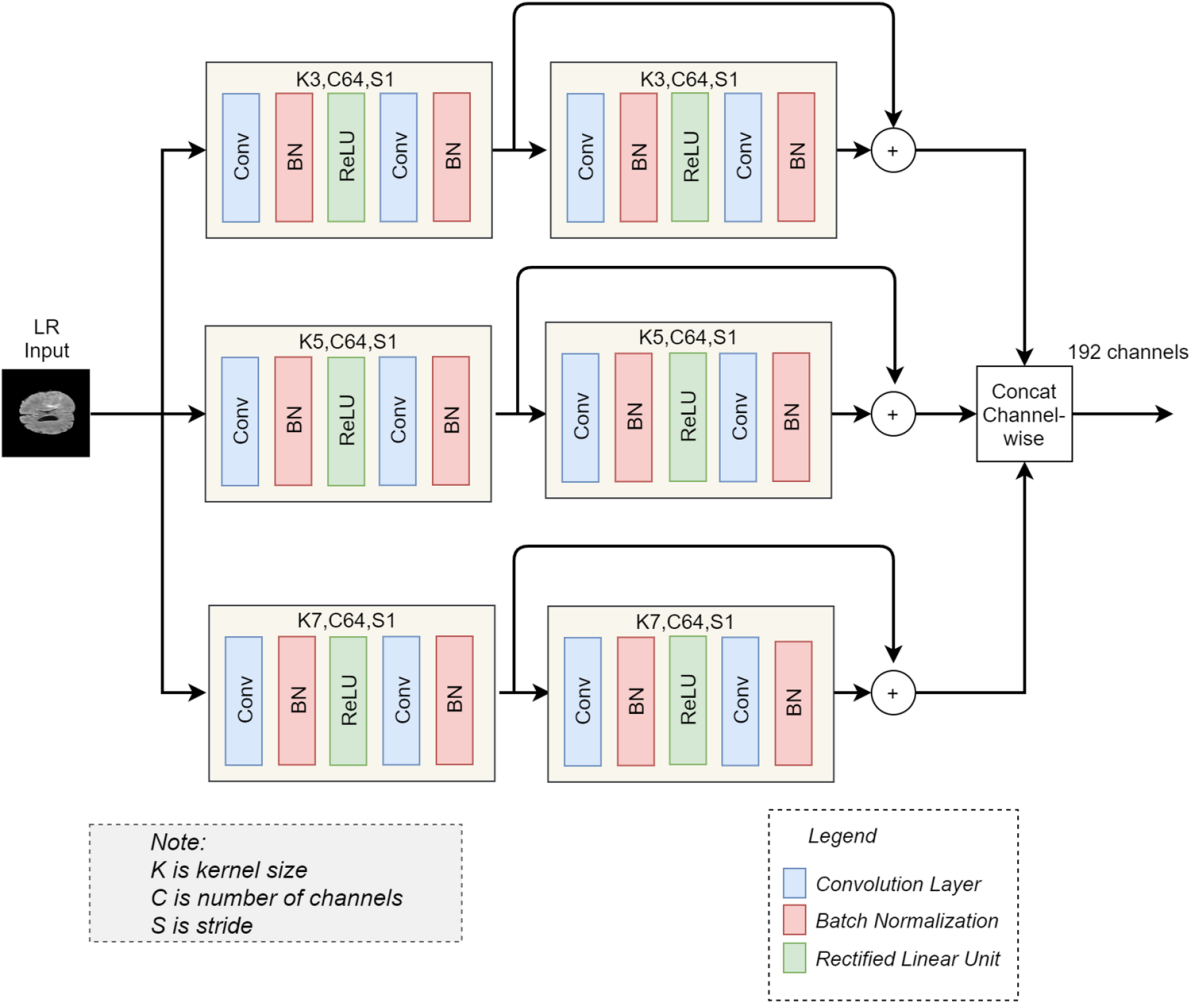
Multi-path upscaling. The figure demonstrates the shallow feature extraction on three different scales. The features are then concatenated channel-wise. K, C and S denotes kernel size, number of channel and stride, respectively.

In this part of the architecture, we use the LR version of the original HR image as an input to the architecture. We calculate basic or shallow features on three different scales by using kernels of sizes 3, 5, and 7, respectively. For feature extraction of each scale, we use two blocks. Each block consists of two convolutional layers, the first convolutional layer in each branch is followed by the batch normalization layer and ReLU activation. In comparison, the second convolutional layer is followed by the batch normalization layer. The number of channels for each pair of convolutional layers is 64. A skip connection is used between the output of the 1st block and the 2nd block. Using the skip connections, the features of the 1st block are added elementwise with the features of the 2nd block. After the feature extraction on different scales, we concatenate (channel-wise) all the three scales features into a single feature vector forming a 192 channels wide features vector as shown in Fig. 2. This feature vector contains the basic or shallow features of the LR image, which will be used as the input to the next step of the proposed architecture. The parameters of this network are shown in Table 1. The extraction of basic or shallow features on three different scales helps in the preservation of the fine details that are very important in medical images. For example, in retinal vessel images, the tiny vessels are important. Hence, it is important to preserve these tiny vessels’ features and map them to HR features. Similarly, for tumor detection in brain MRI, the structure, i.e., edges of the tumor, are important information that should be preserved by preserving the information of tiny edges while mapping LR images to HR images. Therefore, the purpose of using three different scales for basic features extraction is to preserve the fine details on each scale.

**Table 1:** Network parameters for shallow features extraction (read in connection with Fig. 2).

Parameters	Value
No of filters, stride	64, 1
Kernel size	Branch 1 ($3 \times 3$)
	Branch 2 ($5 \times 5$)
	Branch 3 ($7 \times 7$)
Weight initializer (standard deviation)	0.2
Batch normalization (Gamma initializer)	1.0, 0.02
Activation	ReLU

#### Deep features extraction and 2x upscaling

**Figure 3:**
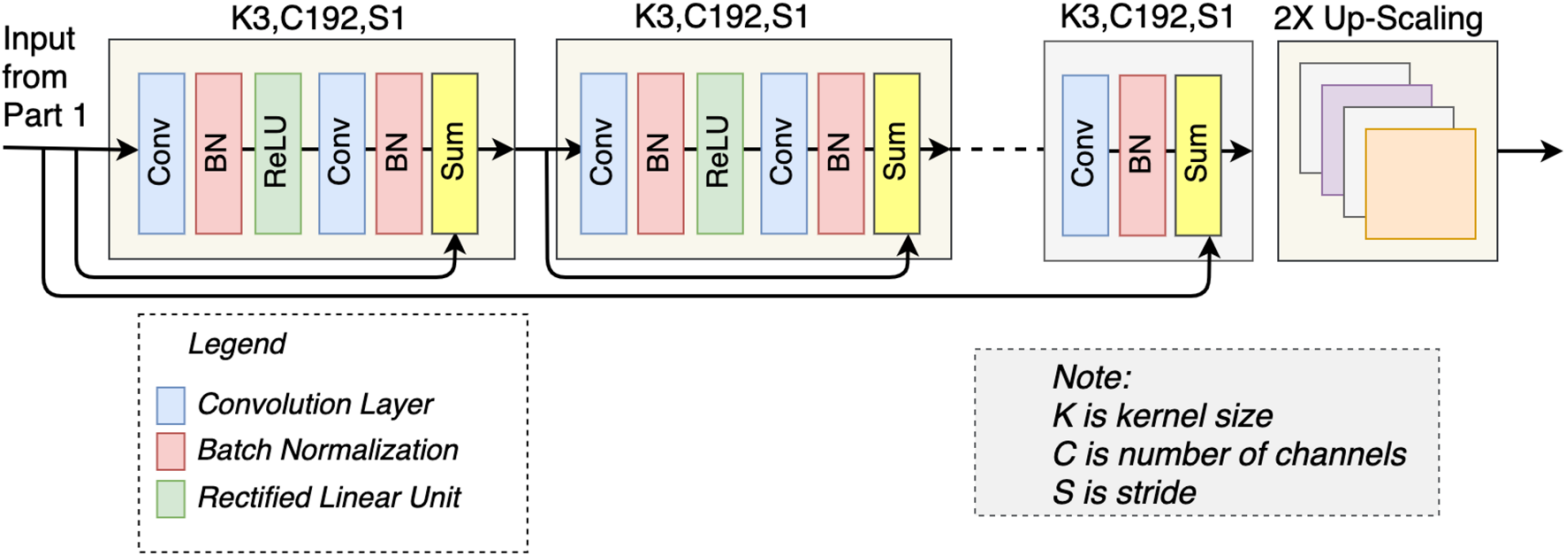
Proposed architecture for the deep features extraction and 2x-upscaling. The dotted line represents 17 blocks, comprising the layers shown in first two blocks. K, C and S represent kernel size, number of Channel and Stride respectively. After this step, we get features map of 2x-upscaled high resolution (HR) image.

In traditional SRGAN^31^, the authors used ResNet34 architecture for deep features extraction with a single pre-residual convolutional layer. Furthermore, each convolutional layer is 64 channels wide. In comparison, we use 192 channels in each convolutional layer. The reason behind using 192 channels in each layer is that we are using the 192 channels wide concatenated output of step 1 as an input in this step of the architecture. As shown in Fig. 3, our ResNet34 architecture consists of 17 blocks, where each block has two convolutional layers. The first convolutional layer is followed by the batch normalization layer and ReLU activation, whereas a batch normalization layer follows the second convolutional layer. The features of each block are added elementwise with the features of the previous block. In the last layer of the ResNet34 architecture, we used kernel size 3 instead of kernel size 9 to overcome the blur effect and preserve more features. In addition, the last layer is added elementwise with the feature map of part 1, as shown in Fig. 3. After deep features extraction, we upscale the extracted features using a sub-pixel convolutional layer. As a result, we have 2x upscaled features of the LR image, which will be used as the input in step 3 of the architecture. The parameters used in this network are listed in Table 2.

**Figure 4:**
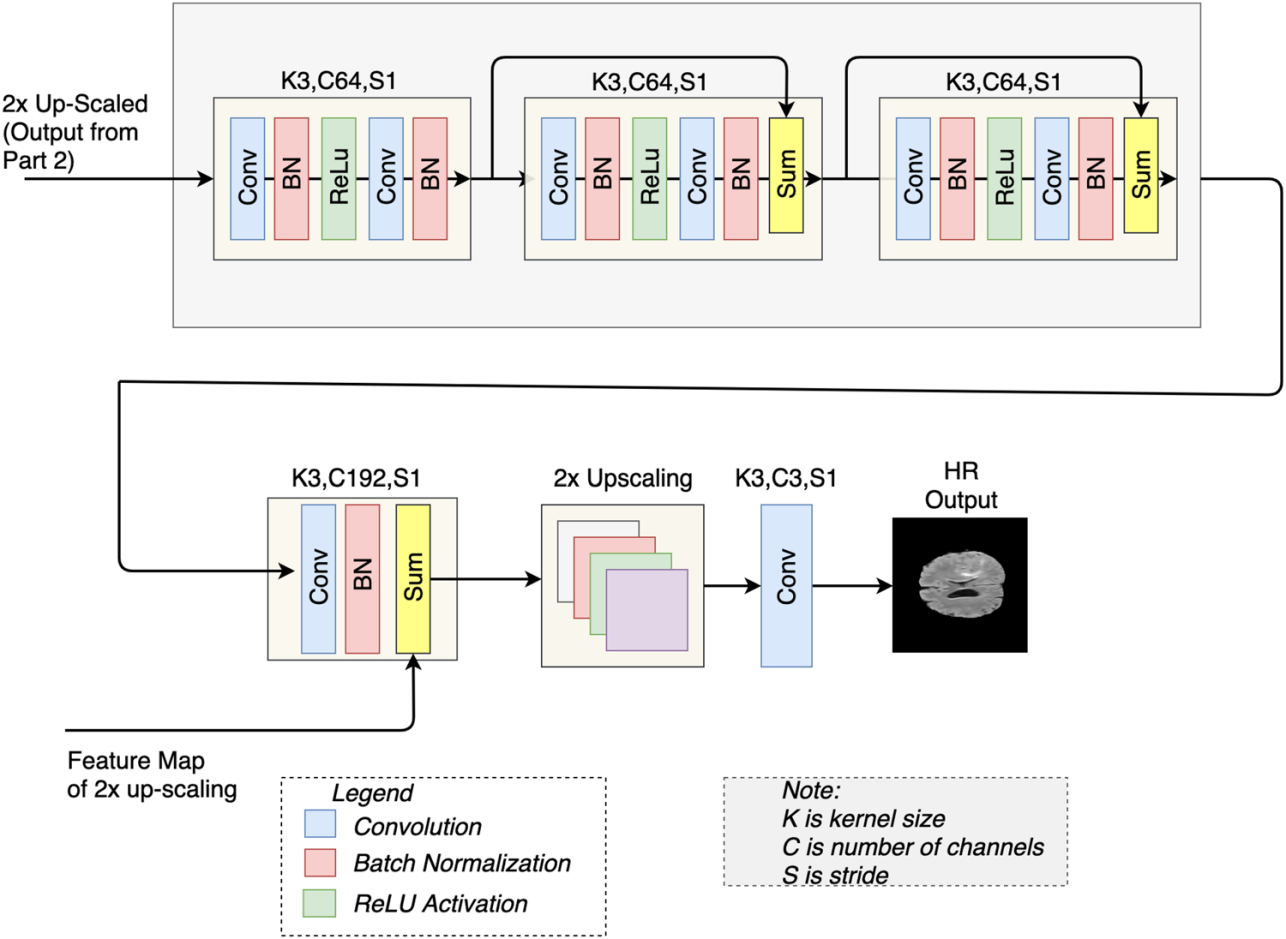
Mini-network: architecture for the mini-network showing the three residual blocks. The features map from Step 2 (see Fig. 3) are added with the output of the mini-network. K, C and S represents the Kernel size, number of Channel and Stride respectively. At the end of this step, we get 4x-upsacled high resolution (HR) image (Predicted HR image).

**Table 2:** Network parameters for the deep features extraction (in Part 2 of Fig. 1).

Parameters	Values
No. of blocks	17
No. of kernels in each layer	192
Kernel size	$3 \times 3$
Stride	$1 \times 1$
Activation function	ReLU
Upscale layer	Sub-pixel layer
Scaling factor	2

#### Features extraction of upscaled version and 2x upscaling

At this step, we have a 2x upscaled version of an LR image. In this step, we calculate features of the 2x upscaled version. We use a mini-network comprising three residual blocks, as shown in Fig. 4. Each residual block has two convolutional layers. The first convolutional layer is followed by the batch normalizationlayer and ReLU activation, whereas a batch normalization layer follows the second convolutional layer. At the end of the mini residual network, a 192-channel wide convolutional layer followed by the batch normalization layer is used. The features map obtained after 2x upscaling in step 2 is elementwise added with the feature map extracted using the mini-network as shown in Fig. 4.

This feature map is then upscaled 2x using a sub-pixel convolutional layer. At the end of step 3, we have a 4x upscaled feature map of the LR image. In the end, a convolutional reconstruction layer is used to map back the 4x upscaled features to the HR image. The parameters used in the mini-network are listed in Table 3. In^31,36^, the authors upscaled the extracted feature map in a single step at the end of the architecture. By upscaling the features in a single step, artifacts may appear in the resultant image due to predicting the wrong information four times in a single step. In our work, we split the process of upscaling into two steps. We extracted the feature of LR image, 2x upscaled the feature map, and rather than again upscaling 2x in the same step, we extracted features of 2x upscaled version and again 2x upscaled the feature map. The significance of this change over other GAN-based methods is discussed in the result section.

**Table 3:** Parameters for the mini-network shown in Fig. 4.

Parameters	Values
No. of blocks	3
No. of kernels in each layer	64
Kernel size	$3 \times 3$
Stride	$1 \times 1$
Activation function	ReLU
Upscale layer	Sub-pixel layer
Scale	2

**Figure 5:**
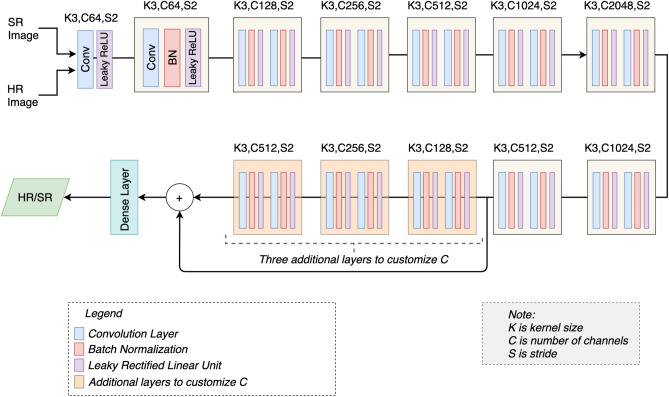
Discriminator network: proposed discriminator network to classify a given input image as an HR or SR image. K shows the kernel size, C shows number of kernels and S shows the stride. Three additional layers having the number of kernels equal to 128, 256 and 512 are added to perform elementwise addition with the parameters of the previous layers.

### Discriminator network

In this work, we also modify the traditional discriminator network of SRGAN^31^. In^31^ the discriminator network consists of 8 convolutional layers, having the number of kernels increasing from 64 to 512, and a single dense layer at the end. Each convolutional layer is followed by the batch normalization layer and ReLU layer. As our generator model is a deep network, hence it requires a strong discriminator in competition, therefore, we propose important modifications in the traditional discriminator. We add more convolutional layers to the discriminator by first increasing the number of kernels in each layer up to 2048 and then reducing the number of kernels progressively down to 512 (see Fig. 5). Thereafter, we have added three additional layers having the number of kernels equal to 128, 256, and 512, respectively. We have elementwise added the output prior to these three layers, and the output of the last 512 kernels layer as shown in Fig. 5. Using skip connection helps to eliminate the vanishing gradient problem.

### Loss functions

As discussed in the literature review, different authors have used different loss functions. In SRGAN^31^, the authors have used three losses that are VGG loss (Content Loss) $L_{VGG}$, generator loss based on cross-entropy *L* *gen*, and mean square error $L_2$ (L2 loss). The mathematical expressions for the above-mentioned losses are expressed in Equation 1, 2, and 3, respectively.

1 $$\begin{aligned} \begin{gathered} L_{V G G}({\bar{I}}, I)= \frac{1}{h w} \sum _{x=1}^{h} \sum _{y=1}^{w}\left( \left( \emptyset _{i, j}\left( I^{HR}\right) _{x, y}-\emptyset _{i, j}\left( G\left( I^{LR}\right) _{x, y}\right) \right) ^{2}\right. \end{gathered} \end{aligned}$$

where $\emptyset _{i, j}\left( I^{HR}\right)$ is the VGG feature vector of the HR image and $\emptyset _{i, j}\left( G\left( I^{LR}\right) \right)$ is the VGG feature vector of the generated image. The variables *h* and *w* represents the height and width of the image, respectively.

2 $$\begin{aligned} L_{g e n}=\sum _{n=1}^{N}-\log D({\bar{I}}) \end{aligned}$$

where $D({\bar{I}} )$ is the output of the discriminator.

3 $$\begin{aligned} L_{2}({\bar{I}}, I) = \frac{1}{hwc} \sum _{i, j, k}\left( {\bar{I}}_{i, j, k} - I_{i, j, k}\right) ^{2} \end{aligned}$$

where ${I}_{i, j, k}$ is the original image and ${{\bar{I}}}_{i, j, k}$ is the generated image. The variables *h*, *w*, and *c* represent the height, width and the number of channels.

In our model, we use all the three losses, along with the absolute difference error (L1 loss) as shown in Eq. (4), which in turn improves the peak signal to noise ratio. L1 loss has the limitation of over smoothing results. This is addressed by using L2 loss along with L1 loss.

4 $$\begin{aligned} L_{1}({\bar{I}}, I) = \frac{1}{hwc} \sum _{i, j, k}\left| {\bar{I}}_{i, j, k} - I_{i, j, k}\right| \end{aligned}$$

where ${I}_{i, j, k}$ is the original image and ${{\bar{I}}}_{i, j, k}$ is the generated image. The variables *h*, *w*, and *c* represent the height, width and the number of channels. The overall loss is then calculated as in Eq. (5):

5 $$\begin{aligned} \text {Overall Loss} = L_{1}({\bar{I}}, I)+L_{2}({\bar{I}}, I)+L_{\text{gen} }+L_{V G G}({\bar{I}}, I) \end{aligned}$$

## Datasets

### Dataset preparation

All experiments are carried out on four publicly available datasets. These include two fundoscopy datasets of retinal images, i.e., DRIVE^37^ and STARE^38^, a dermoscopy dataset of skin cancer images (ISIC)^39^, a dataset of brain MRI (BraTS 2018), and a dataset of 2D cardiac ultrasound images^41^. The preparation details for each dataset for our experiments are given below.

#### Retinal images dataset

Retinal images datasets include two datasets, DRIVE and STARE. DRIVE dataset contains 40 retinal images, where 20 images are training images while 20 images are test images. STARE dataset includes 397 images. We randomly select 20 images from the STARE dataset as test images. The remaining images and 20 training images from the DRIVE dataset are used as training images. The randomly selected 20 images from STARE and 20 test images from the DRIVE dataset are used as testing images for our experiments. The original resolution of each image in DRIVE and STARE is 565x584 and 700$\times$605, respectively. However, for our experiment, each image is resized to 512$\times$512 resolution. We used these resized images as HR images. To obtain LR images, each HR image is downsampled four times to 128$\times$128 resolution. A batch size of 2 is used for the retinal image super-resolution experiment.

#### Skin cancer dataset

ISIC skin cancer dataset contains 540 skin dermoscopy images. Each image in the dataset is of a different resolution. For our experiments, we resized each image to 512$\times$512 and used these images as HR images. We further downsampled the images 4 times to 128$\times$128 resolution for use as LR images. We used 500 images for training and 40 images for testing. A batch size of 2 is used for the skin cancer image super-resolution experiment.

#### Brain tumor MRI dataset

BraTS 2015 dataset includes Brain Tumor MRI images developed for brain tumor segmentation task. This dataset contains two types of tumor data, i.e., high-grade glioma (HGG) and low-grade glioma (LGG). Four different modalities data are provided for each type. Each image is a 3D volume and is available in Nifti format. For our experiments, we extracted 2D slices from each 3D volumetric image. Each slice has a 240$\times$240 resolution. In this work, we use 240$\times$240 2D slices as ground truth HR image and downsampled the HR slice 4 times to 60$\times$60 resolution, which is used as an LR image. We used 400 images for training and 30 images for testing. A batch size of 4 is used for the brain MRI image super-resolution experiment.

#### Cardiac ultrasound images dataset

The CAMUS (Cardiac Acquisition for Multi-structure Ultrasound Segmentation) dataset^41^ contains cardiac 2-chamber and 4-chamber 2D ultrasound images. The dataset is publicly available and contains 500 cardiac ultrasound images. Each image has a different resolution, and all of them are larger than 1024$\times$512. Therefore, each image is re-scaled to a resolution of 1024$\times$512 for use as ground truth. To obtain the LR version, each image is downsampled 4 times to 256$\times$128 resolution. For the CAMUS dataset, we use 400 images for training and 100 images for testing, and a batch size of 2.

## Results and discussion

### Evaluation metrics

The following evaluation measures were used to compare the performance of the proposed work with other super-resolution methods.

#### Peak signal-to-noise ratio

Peak signal-to-noise ratio (PSNR) is used to measure the quality of the reconstructed image. PSNR is the ratio of the maximum possible power of a signal to the noise that affects the quality of the signal. Mathematically, PSNR is shown in Eq. (6).

6 $$\begin{aligned} {\text {PSNR}} = 10 \cdot \log _{10}\left( \frac{\max (I)^{2}}{M S E}\right) \end{aligned}$$

where, *I* is the image and *MSE* is mean square error (MSE). The lower the MSE, the higher will be PSNR. MSE is shown in Eq. (7).

7 $$\begin{aligned} M S E=\frac{1}{hw} \sum _{x=0}^{h-1} \sum _{y=0}^{w-1}(I(x, y)-{\bar{I}}(x, y))^{2} \end{aligned}$$

where, *I*(*x*, *y*) is the original image and ${\bar{I}}(x, y)$ is generated image. The variables *h* and *w* represent the height and width of the image.

#### Structure similarity index

The structural similarity index (SSIM) presents the degradation of image quality caused by processing the image such as image compression or image up-sampling or down-sampling. It shows the similarity of structure between the actual image and the reconstructed image. SSIM can mathematically be represented as in Eq. (8).

8 $$\begin{aligned} {\text {SSIM}}(I, {\bar{I}})=\frac{\left( 2 \mu _{I} \mu _{{\bar{I}}}+c 1\right) \left( 2 \sigma _{I {\bar{I}}}+c 2\right) }{\left( \mu _{I}^{2}+\mu _{{\bar{I}}}^{2}+c 1\right) \left( \sigma _{I}^{2}+\sigma _{{\bar{I}}}^{2}+c 2\right) } \end{aligned}$$

where $\mu _{I} \mu _{{\bar{I}}}$ is the average of the original image *I* and the reconstructed image ${\bar{I}}$, respectively, $\sigma _{I}$ and $\sigma _{{\bar{I}}}$ are the variances of the original image *I* and the reconstructed image ${\bar{I}}$, respectively, and *c*1 and *c*2 are the two variables to stabilize the equation.

The results for each dataset are discussed below.

### Results on retinal images

The PSNR and SSIM scores of the proposed architecture are compared with the traditional SRGAN^31^ and Bicubic interpolation method. Table 4 shows the score of PSNR and SSIM on the retinal images’ dataset for each method. For the STARE dataset, our method improves the PSNR score by 4.95 dB compared to traditional SRGAN and by 11.52 dB compared to the Bicubic interpolation method. SSIM score is increased by 4 percent compared to traditional SRGAN and 6 percent compared to the Bicubic interpolation method. The experiment is repeated ten times to find the standard deviation. Similar improvement is shown while testing on the DRIVE dataset.

**Table 4:** Results on retinal images dataset.

Algorithm	STARE		DRIVE	
	PSNR (dB) ± std	SSIM ± std	PSNR (dB) ± std	SSIM ± std
Bicubic interpolation	27.05 ± 2.25	0.85 ± 0.21	25.2 ± 2.12	0.86 ± 0.17
SRGAN^31^	33.62 ± 3.16	0.87 ± 0.27	34.22 ± 3.51	0.88 ± 0.20
Proposed method	**38.57 **± **3.45**	**0.91 **±** 0.32**	**37.72 ** ±** 3.1**	**0.91 **±** 0.27**

Significant values are in [bold].

**Figure 6:**
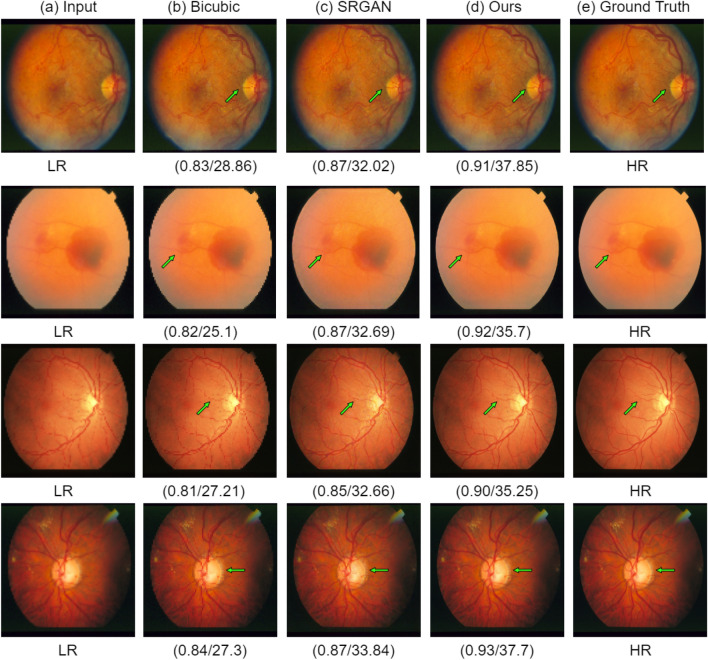
Results of super resolution for different images of STARE dataset. The column (**a**) has LR images (128$\times$128 but scaled for uniform display), column (**b**) shows Bicubic interpolation SR result, column c shows output HR images for SRGAN^31^, column d shows output HR images for the proposed architecture, column e shows ground truth HR images (512$\times$512). Each row illustrates results of a single image. The corresponding (SSIM/PSNR) scores are mentioned below each image. Green arrows illustrate the degradation or improvement caused by each algorithm with respect to the ground truth HR image.

**Figure 7:**
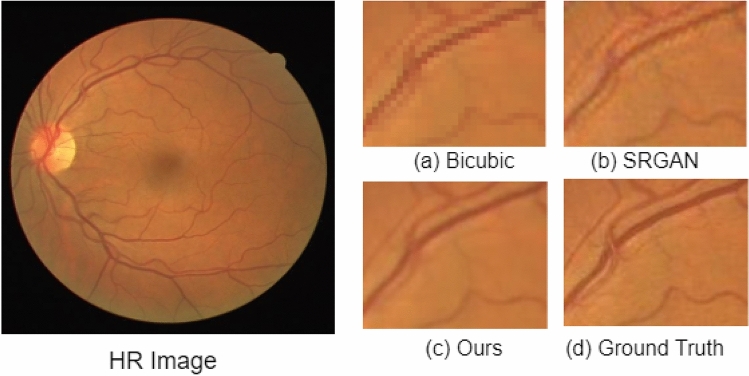
Small region results for an image from DRIVE dataset. The sub-region for (**a**) Bicubic interpolation. (**b**) SRGAN^31^. (**c**) Proposed architecture. (**d**) Ground truth HR image.

**Figure 8:**
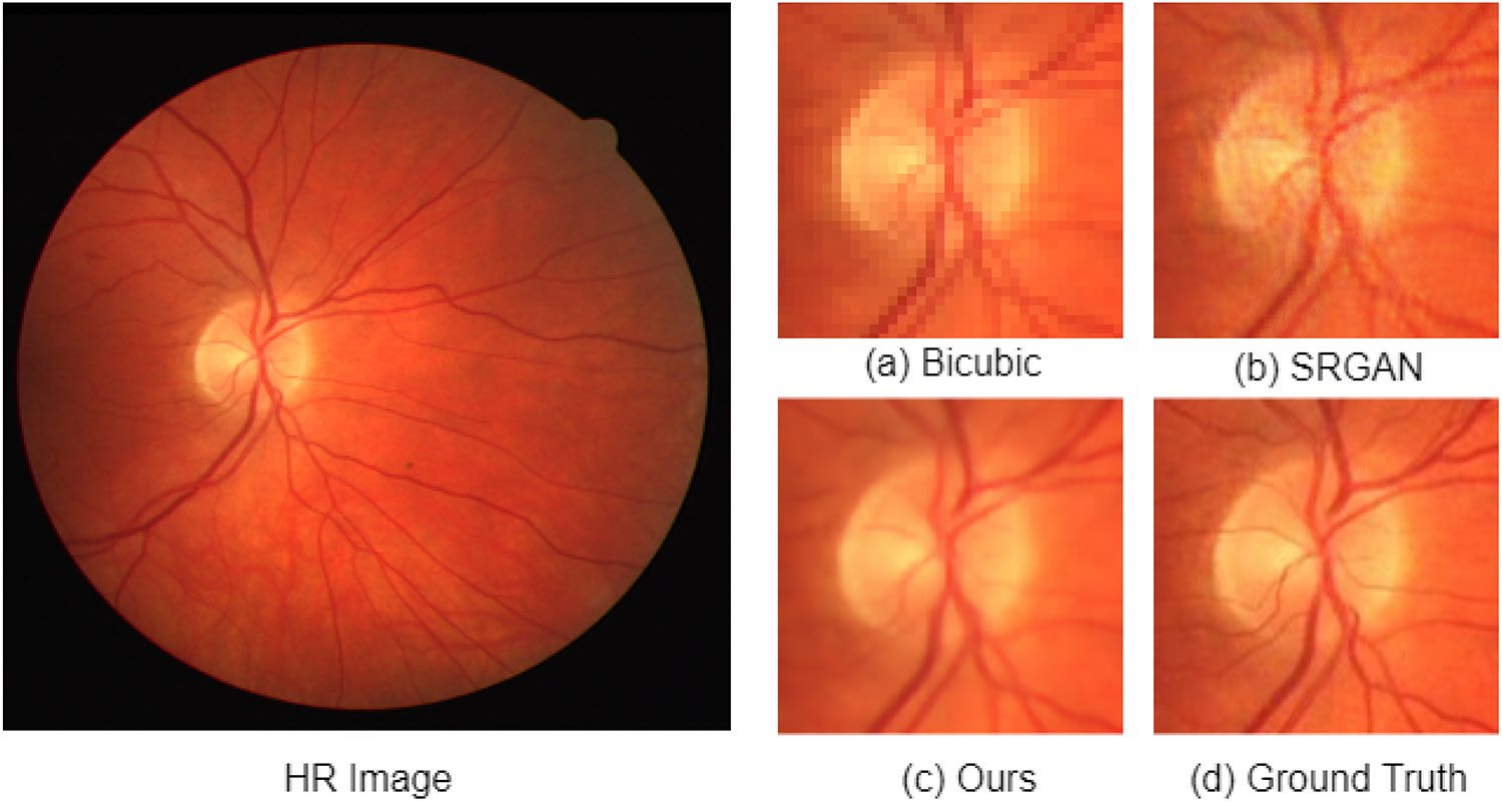
Small region results for an image from DRIVE dataset. The sub-region for (**a**) Bicubic interpolation. (**b**) SRGAN^31^. (**c**) Proposed architecture. (**d**) Ground truth HR image.

The SR results on the STARE dataset are shown in Fig. 6. In Fig. 6, each row shows the result for a single image. The LR image, the interpolation-based SR results, SRGAN^31^ results, proposed architecture results, and the corresponding ground truth HR images are shown in Fig. 6a–e, respectively. The green arrow shows the area where there exists the degradation caused by each method. The interpolation-based method degrades the structure by making it very blurry, increasing the mean square error. The SRGAN^31^ method removes the blurriness in the image but adds a lot of noise which can be seen near the optic disk. In comparison, the proposed architecture preserves the structure of the vessels and removes the noise making the image smooth. In Figs. 7 and 8, we have extracted small regions from two images of the DRIVE dataset to show the image quality degradation caused by each algorithm. The proposed architecture produces more realistic HR images compared to the other two methods.

### Results on skin images

**Figure 9:**
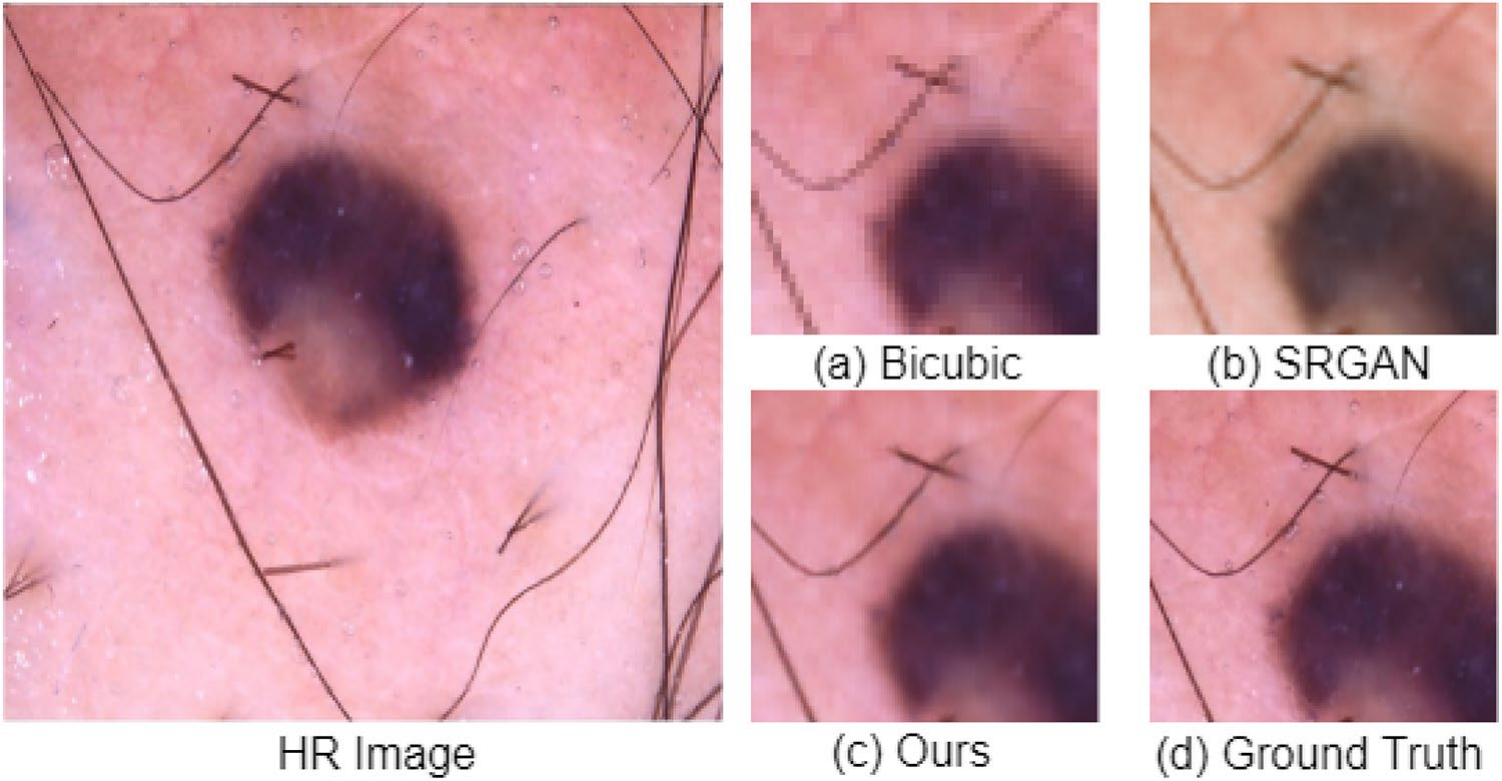
Small region results on image from ISIC skin cancer dataset. The sub-region for (**a**) Bicubic interpolation. (**b**) SRGAN^31^. (**c**) Proposed architecture. (**d**) Ground truth HR image.

**Figure 10:**
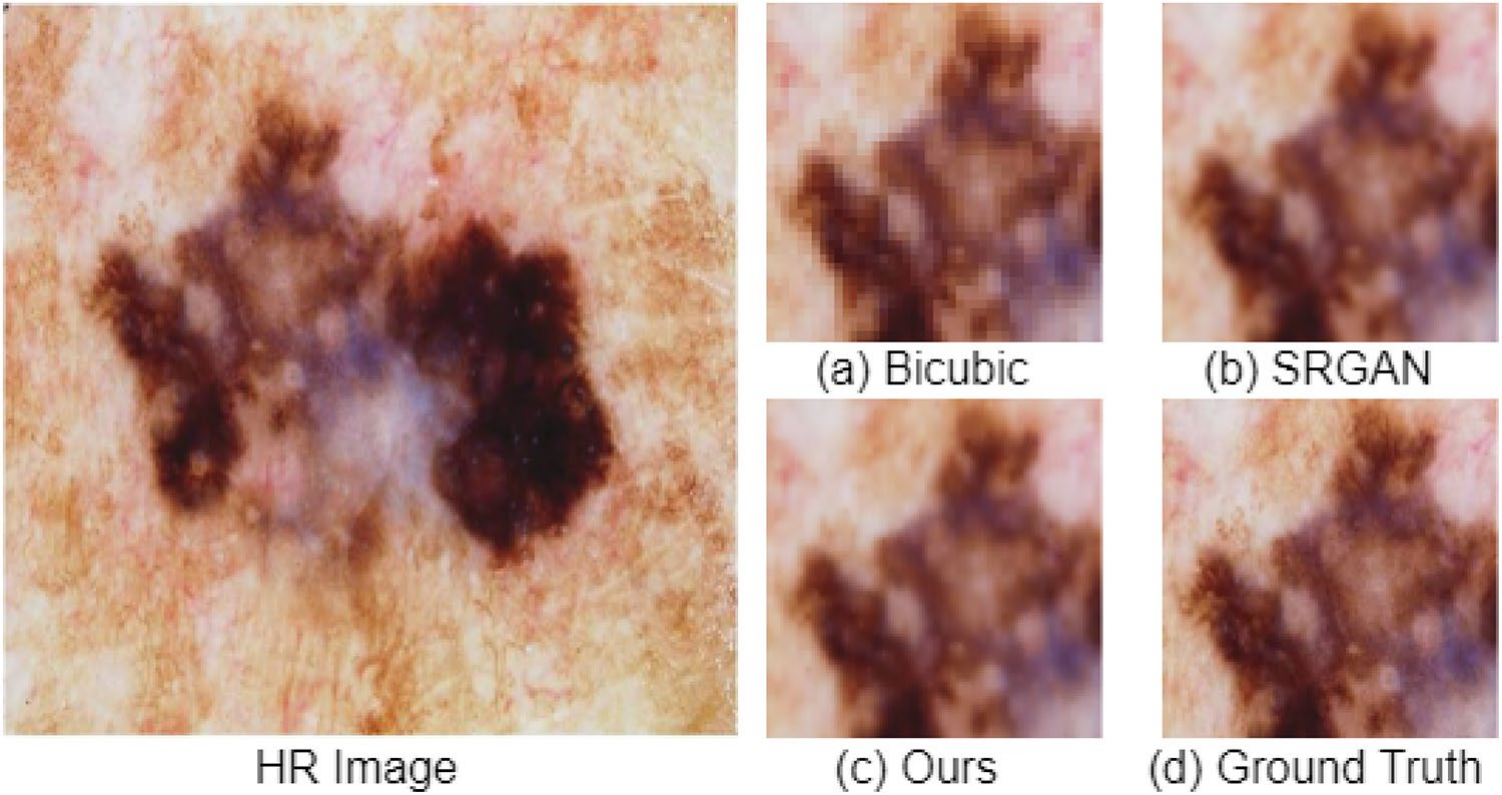
Small region results on image from ISIC skin cancer dataset. The sub-region for (**a**) Bicubic interpolation. (**b**) SRGAN^31^. (**c**) Proposed architecture. (**d**) Ground truth HR image.

The PSNR and SSIM scores for the skin dataset are shown in Table 5. The proposed architecture improved PSNR by 3.11 dB and SSIM by 4 percent compared to SRGAN, and PSNR by 9.27 dB, and SSIM by 2 percent compared to the bicubic interpolation. The experiment is repeated ten times for each method to report the standard deviation. In Figs. 9 and 10, we have extracted small regions from resultant images to show the qualitative improvement of our model. As shown in Figs. 9b and 10b, the SRGAN does not preserve colors correctly, and hence SSIM is heavily affected, while the bicubic method adds artifacts and blurriness in the resultant image (see Fig. 9a). The output of the proposed architecture is shown in Figs. 9c and 10c, which are qualitatively very similar to the ground truth HR images in Figs. 9d and 10d, respectively.

**Table 5:** Results on skin images dataset.

Algorithm	PSNR (dB) ± std	SSIM ± std
Bicubic interpolation	25.15 ± 2.3	0.88 ± 0.12
SRGAN^31^	31.31 ± 2.7	0.86 ± 0.21
Proposed	**34.42 **±** 2.0**	**0.90 ** ± ** 0.14**

Significant values are in [bold].

### Results on brain MRI images

**Figure 11:**
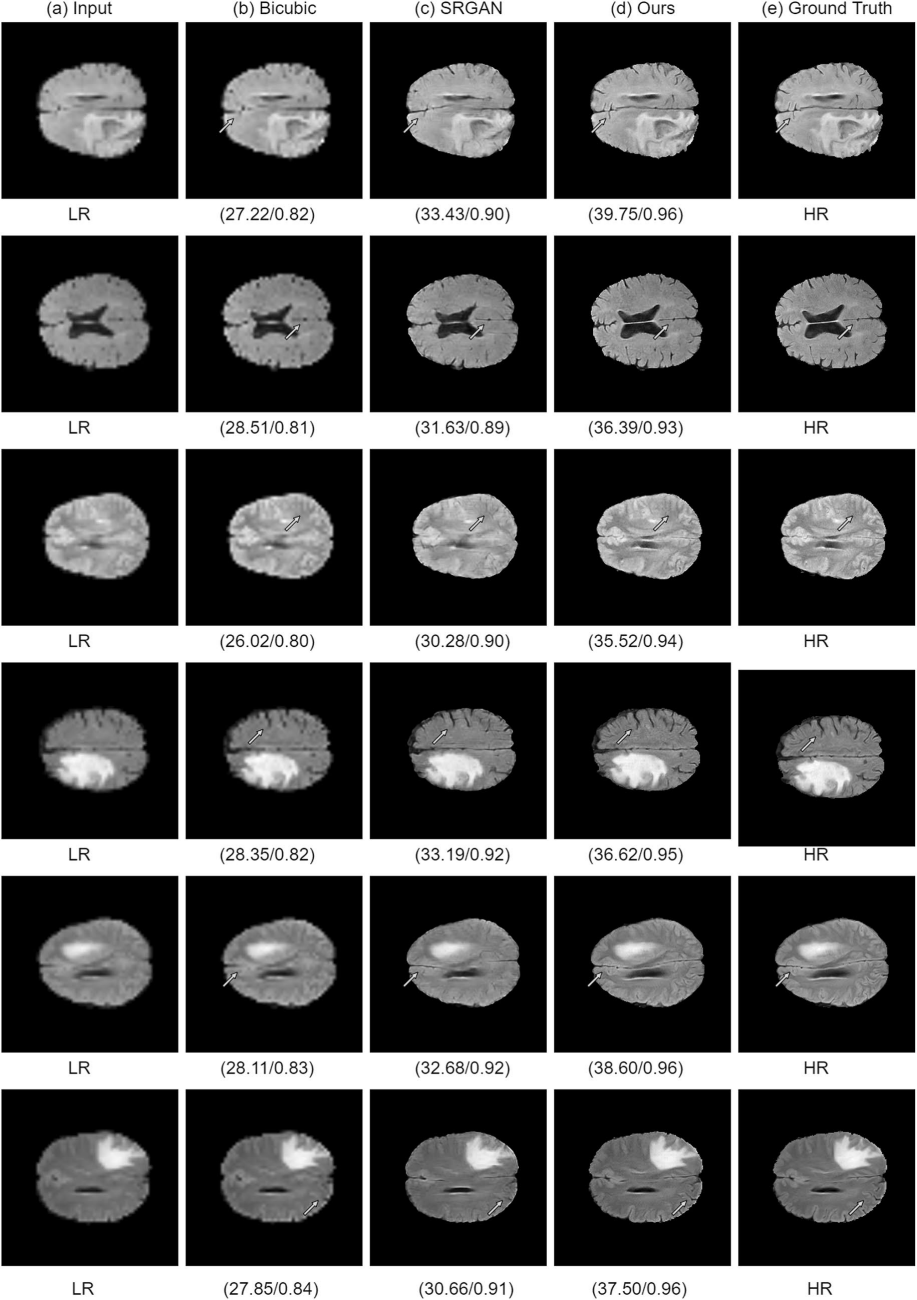
Results of super resolution for different images of BRaTS dataset. Column (**a**) shows the LR images (60$\times$60, scaled for uniform display), column (**b**) shows results for Bicubic interpolation, column (**c**) shows results for SRGAN^31^, column (**d**) shows the results for the proposed architecture, column (**e**) shows ground truth HR images ($240 \times 240$). Each row illustrates result of a single image. The corresponding (SSIM/PSNR) scores is mentioned below each image. Green arrows illustrate the degradation or improvement caused by each algorithm with respect to the ground truth HR image.

**Figure 12:**
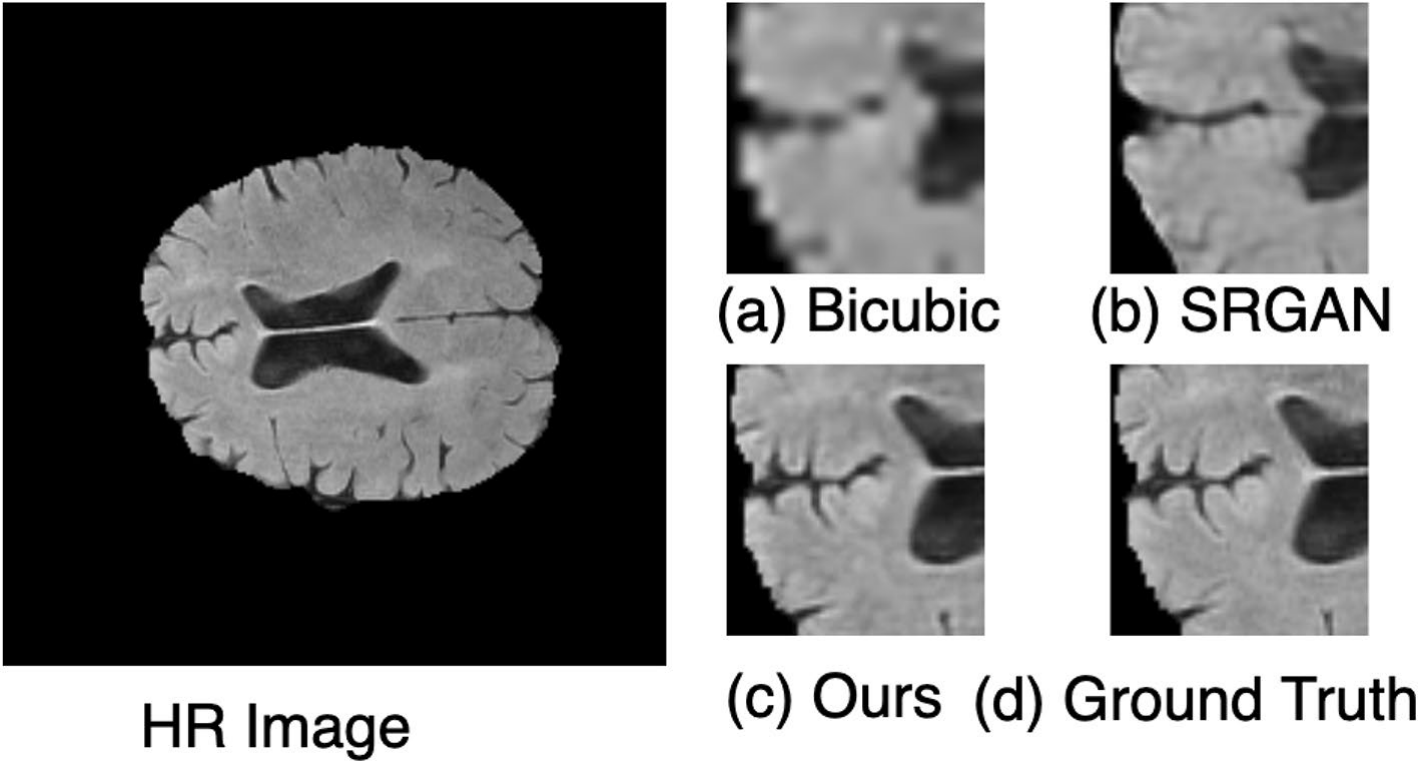
Small region results on MRI image. The sub-region for (**a**) Bicubic interpolation. (**b**) SRGAN^31^. (**c**) Proposed architecture. (**d**) Ground truth HR image.

The PSNR and SSIM scores for the BraTS dataset are shown in Table 6. The proposed method improved PSNR and SSIM by 9.31 dB and 7 percent, respectively, compared to bicubic interpolation and 6.35 dB and 5 percent, respectively, compared to the SRGAN method. The visual results of each method are shown in Fig. 11. Each row contains the SR results of a single image. The arrow shows the degradation caused by each method. It can be seen from Fig. 11b that the interpolation-based method produces blurry results. On the other hand, the SRGAN^31^ does not preserve the actual structure and adds noise to the resultant image, as shown in Fig. 11c. In comparison, the proposed architecture in Fig. 11d generates SR output images that are very close to the images in Fig. 11e, i.e., corresponding HR images. In Fig. 12, we have extracted a small region from an image and compared each method. These figures show that the proposed method generates more realistic HR images than SRGAN^31^ and the interpolation-based method.

**Table 6:** Results on brain MRI images.

Algorithm	PSNR (dB) ± std	SSIM ± std
Bicubic interpolation	29.52 ± 2.2	0.88 ± 0.15
SRGAN^31^	32.48 ± 2.5	0.90 ± 0.22
Proposed	**38.83 **± ** 2.1**	**0.95 **±** 0.17**

Significant values are in [bold].

Furthermore, in Table 7, we have compared the PSNR and SSIM of the proposed method with other state-of-the-art methods for the BraTS dataset. The results of 2D FSRCNN^42^ and 3D FSRCNN are taken from 3D DCSRN^43^. The results of SRCNN^14^, VDSR^44^, and FSCWRN^44^ are taken from CSN^45^. The results for SRGAN^31^ are generated by us. Table 7 shows that the proposed method outperformed other methods.

**Table 7:** Results on BraTS dataset: additional comparison with state-of-the-art methods.

Algorithms	Publication year	PSNR (dB) ± std	SSIM ± std
SRCNN^14^	2015	29.90	0.89
VDSR^44^	2016	30.57	0.89
2D FSRCNN^42^	2016	31.55 ± 1.7	0.88 ± 0.11
3D FSRCNN^43^	2016	33.86 ± 1.7	0.91 ± 0.12
SRGAN^31^	2017	33.48 ± 2.1	0.90 ± 0.20
3D DCSRN^43^	2018	35.05 ± 1.8	0.93 ± 0.11
FSCWRN^44^	2018	30.96	0.90
CSN^45^	2019	31.23	0.90
Proposed	–	**38.83 **±** 2.1**	**0.95 **±** 0.17**

Significant values are in [bold].

### Results on ultrasound images

**Figure 13:**
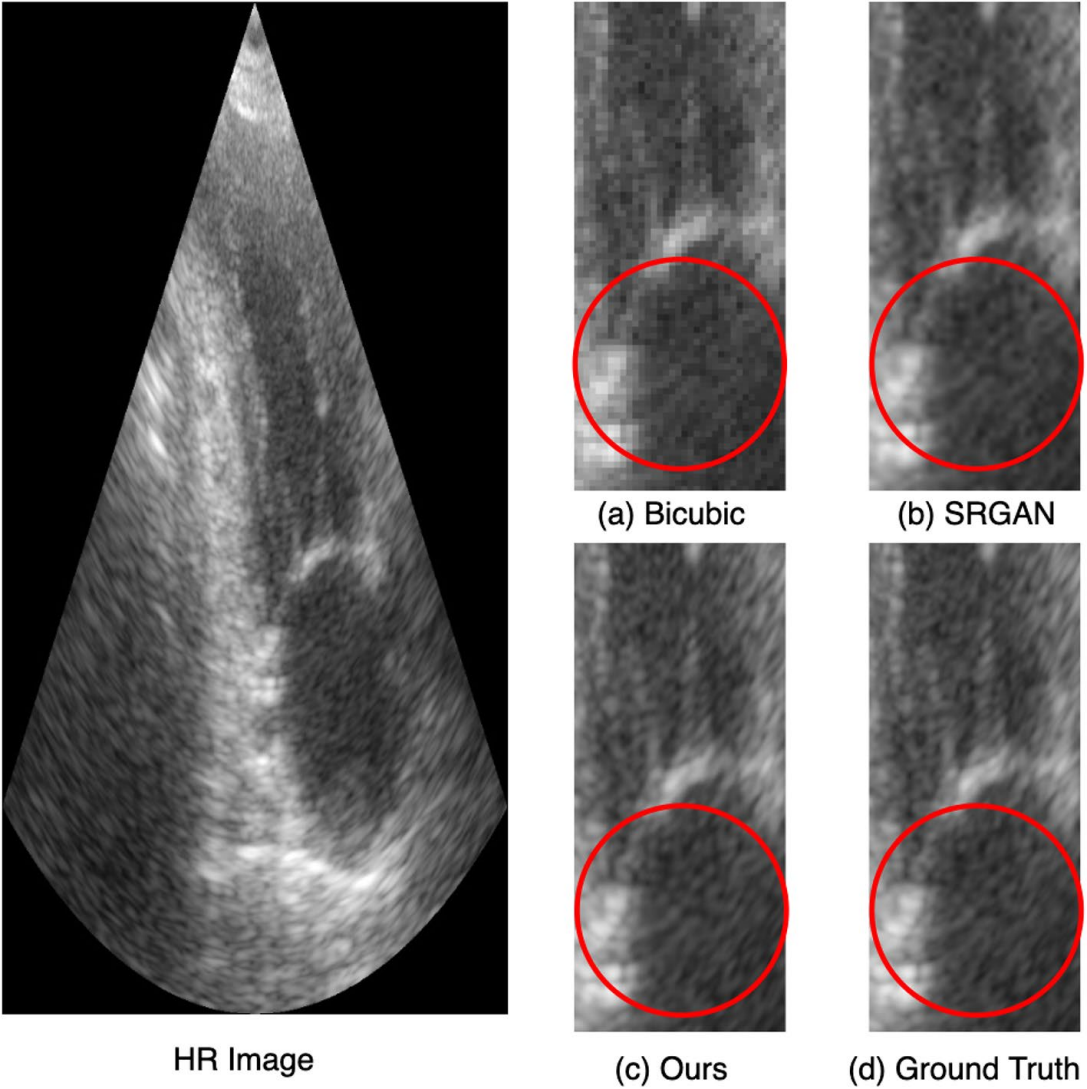
Small region results on cardiac ultrasound image. The sub-region for (**a**) Bicubic interpolation. (**b**) SRGAN^31^. (**c**) Proposed architecture. (**d**) Ground truth HR image.

The PSNR and SSIM scores for the CAMUS dataset of cardiac ultrasound images are shown in Table 8. The proposed architecture improved PSNR by 1.65 dB and SSIM by 2 percent compared to SRGAN, PSNR by 3.9 dB, and SSIM by 6 percent compared to bicubic interpolation. The experiment is repeated ten times to obtain the standard deviation. To better visualize the qualitative improvement of the proposed model, we have extracted small regions from the resultant images to identify the qualitative improvement of the proposed model, as shown in Fig. 13. The bicubic interpolation output shown in Fig. 13a has blur effects, and the original details are not preserved. Similarly, in Fig. 13b, the output of SRGAN has a dark shade though less explicit, compared to Fig. 13d, i.e., ground truth HR image. Finally, the output of the proposed architecture is shown in Fig. 13c, which is very similar to the ground truth HR image in Fig. 13d.

**Table 8:** Results on ultrasound images.

Algorithm	PSNR (dB) ± std	SSIM ± std
Bicubic interpolation	33.21 ± 1.5	0.89 ± 0.20
SRGAN^31^	35.46 ± 2.1	0.93 ± 0.20
Proposed	**37.11 **±** 1.7**	**0.95 ** ±** 0.23**

Significant values are in [bold].

### Results on noisy data

**Figure 14:**
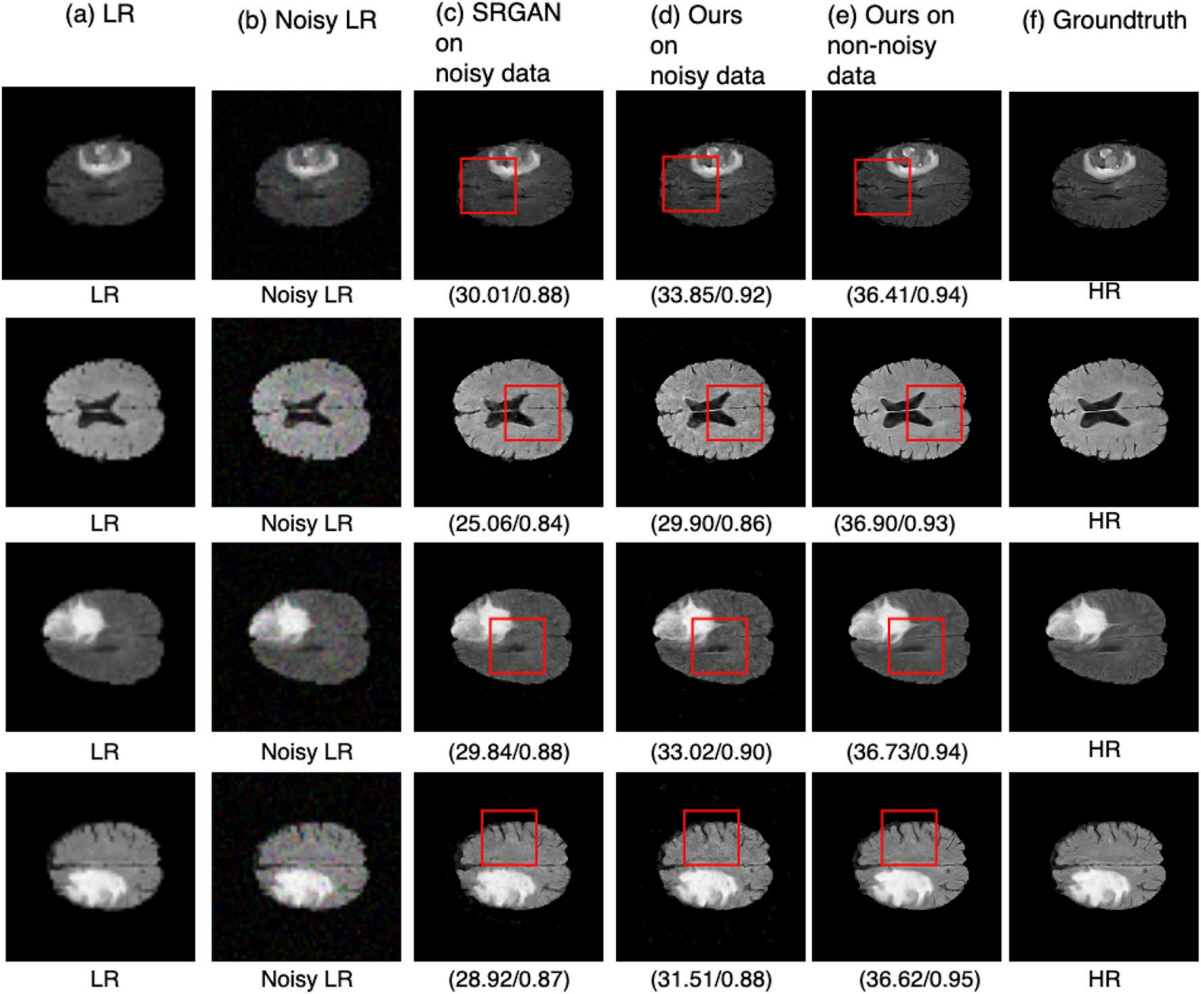
Results of super resolution for noisy MRI images. Column (**a**) shows the LR images ($60 \times 60$ but scaled for uniform display), Column (**b**) shows the noisy LR images ($60 \times 60$ but scaled for uniform display), column (**c**) shows the results for SRGAN^31^, column (**d**) shows the results for the proposed architecture, column (**e**) shows the results of proposed method on non-noisy images, column (**f**) shows ground truth HR images ($240 \times 240$). Each row illustrates result of a single image. (SSIM/PSNR) score is shown for each image. Red box illustrates the degradation or improvement caused by each algorithm w.r.t ground truth HR image.

**Figure 15:**
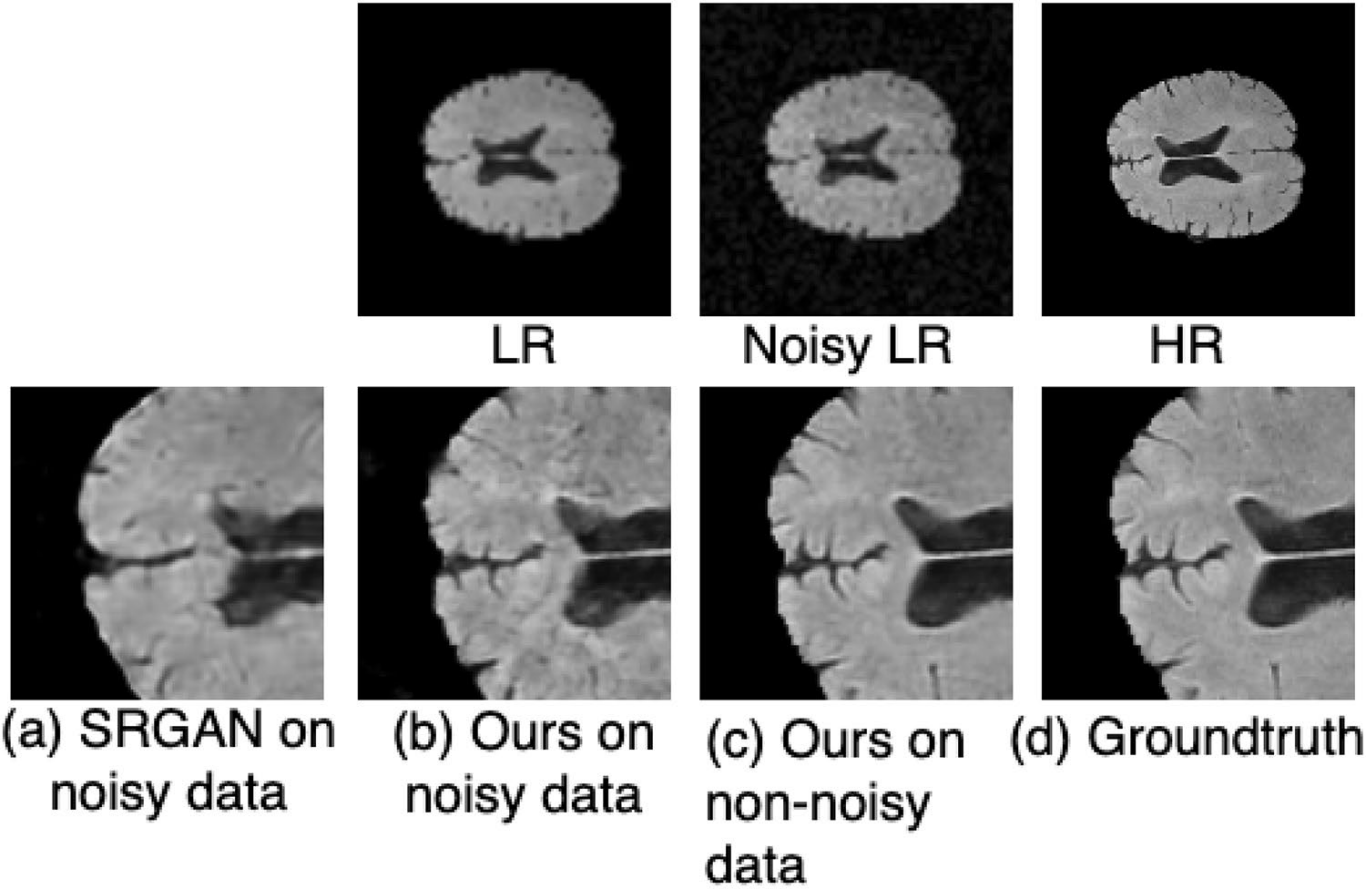
Small region results for noisy data. Top Row shows LR image, the noisy LR image and ground truth HR image. Bottom row contains the extracted small region results. (**a**) Shows the SRGAN results. (**b**) Shows the results of the proposed method on noisy image. (**c**) Shows the results of the proposed method on original image (non noisy) image, and (**d**) shows the corresponding ground truth HR image. Images re-scaled for better display purpose.

The proposed model is also evaluated using noisy brain MRI images. Gaussian noise with zero mean and variance of 0.01 is added to the brain MRI images. The SSIM and PSNR scores for both SRGAN and the proposed methods used on noisy data are shown in Table 9. The proposed architecture improved SSIM by 4 percent and PSNR by 4.85 dB compared to SRGAN^31^. As shown in Fig. 14, the proposed method outperformed the SRGAN^31^ method with a noticeable improvement. In Fig. 14, each row represents the results for a single image. To further identify the differences, small regions from the resultant images are shown in Fig. 15. The first row in Fig. 15 shows the LR image, the noisy LR, and the corresponding HR image. The SRGAN^31^ method does not preserve the structure of the image and hence causes noticeable degradation as shown in Fig. 15a, while the proposed method produces better results on noisy images shown in Fig. 15b. he proposed method result on noisy image is very close to the result on non-noisy image shown in Fig. 15c and the ground truth HR image shown in Fig. 15d.

**Table 9:** Results on noisy images.

Algorithm	PSNR (dB) ± std	SSIM ± std
SRGAN on noisy data	28.58 ± 1.4	0.87 ± 0.23
SRGAN on non-noisy data	32.48 ± 2.5	0.90 ± 0.22
Proposed method on noisy data	**33.43 **±** 2.1**	**0.91 ** ±** 0.31**
Proposed method on non-noisy data	**38.83 **±** 2.1**	**0.95** ± ** 0.17**

Significant values are in [bold].

### Results analysis

The results on the four datasets show significant improvement of performance achieved by the proposed method compared to other SR methods, particularly the interpolation-based methods and the SRGAN^31^. In SRGAN^31^, the authors extracted basic or shallow features using a single scale, i.e., kernel size 3. However, the features of large scale are missed by kernel size 3. Therefore, in part 1 of our proposed architecture, we extracted the shallow features on three different scales, i.e., kernel size 3, 5, and 7. Subsequently, we have concatenated all features into a single feature vector, as shown in Fig. 2. The effect of this can be seen, for instance, in Fig. 12b, where the SRGAN missed the important features, while in Fig. 12c, the proposed architecture preserves the correct structure of the image while generating the HR image.

Furthermore, the second important proposed change in the architecture of SRGAN^31^ is that we have used the idea of progressive upscaling. In SRGAN^31^, the authors performed upscaling in a single step, i.e., after the feature extraction. The limitation of performing upscaling is that it does not generate true colors while generating an HR image. In our proposed work, we have extracted features of an LR image, then performed 2x upscaling, and extracted the features of the upscaled version, and again performed 2x upscaling. The advantage of this change can be seen in Figs. 9 and 10. In Figs. 9b and 10b, the SRGAN architecture does not preserve the actual colors in the generated HR images, while in Figs. 9c and 10c, the proposed architecture generates realistic colors very similar to the ground truth HR images in Figs. 9d and 10d.

The third proposed change is that we have added an extra loss function to the loss functions used in SRGAN^31^. We have used L1 loss, i.e., mean absolute error given in Eq. (4). The overall loss function used in the proposed architecture is shown in Eq. (5). L1 loss penalizes large errors. Therefore, using L1 loss in addition to the other three losses generates more realistic and smooth images. The significance of using L1 loss can be seen in Figs. 7 and 8. As shown in Figs. 7b and 8b, the SRGAN generates the HR image with noise in it. The noise can be seen around the vessel in Fig. 7b and near the optic disc in Fig. 8b. In Figs. 7c and 8c, the proposed architecture generates smoother HR images similar to the corresponding HR image shown in Figs. 7d and 8d, respectively.

The results presented in this work are obtained on an Nvidia GTX 1080 Ti GPU and the code is implemented in Tensorflow 2.0.

### Ablation studies

**Figure 16:**
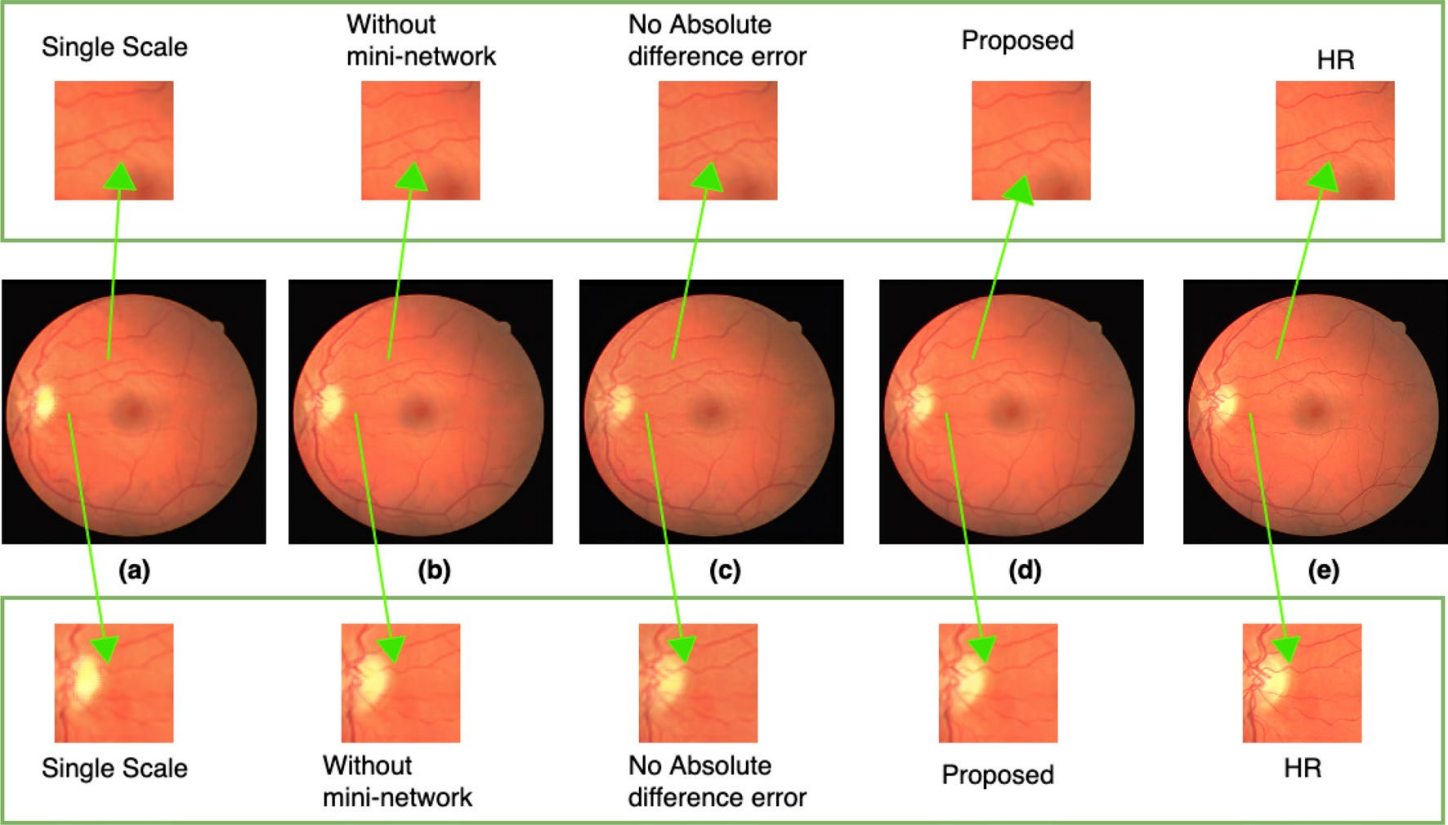
Results for ablation studies. The upper and lower rows show the extracted small region. (**a**) Represents the resultant SR image with single scale features extraction. (**b**) Represents the resultant SR image for experiment without the mini-network. (**c**) Represents the resultant SR image for experiments after removing the absolute difference error term from the loss function. (**d**) Represents the resultant SR image for experiment with the overall proposed method. (**e**) Represents the corresponding HR ground truth image. Arrows point to the extracted small regions to highlight the regions where difference occurs.

To show the effectiveness of the various modules of the proposed method, we carried out an ablation study on the retinal images from the DRIVE dataset. We performed ablation using three different steps. In the first step, we made changes to the feature extraction module. In this step, we extracted features on a single scale only, i.e., using kernel size 3. By doing so, we recorded that the SSIM and PSNR scores were reduced by 3.2 dB and 3 percent, respectively. In the second step, we made changes to the mini-network module of the proposed method. We removed the mini-network between the two subpixel convolution layers in this step. By doing so, we observed a 4.62 dB decrease in the PSNR and a 5 percent decrease in SSIM score. In the third step, we removed the additional error function, i.e., the absolute difference error of Eq. (4). By removing the additional loss function, the PSNR and SSIM scores decreased by 6.58 dB and 8 percent, respectively. The scores for each experiment of the ablation study are shown in Table 10. To visualize the difference, we have extracted small regions from the resultant images as shown in Fig. 16. From Fig. 16a, it is clear that the experiment using a single scale features extraction resulted in a low-quality image. The degraded result for the second experiment “without mini-network” is shown in Fig. 16b. Figure 16c shows the resultant image of the third experiment, i.e., by removing the absolute difference error term from the loss function. The noise is clearly seen in the small extracted patch. The result of the proposed method incorporating all the modules of the proposed method is shown in Fig. 16d, which produces a better SR image that is very close to the HR ground truth image shown in Fig. 16e.

**Table 10:** Ablation study for the proposed method using retinal images.

Ablation experiment	PSNR (dB) ± std	SSIM ± std
Single scale features extraction	34.5 ± 1.3	0.88 ± 0.15
Remove mini-network	33.10 ± 0.9	0.86 ± 0.21
Remove the absolute difference error loss function	31.04 ± 2.1	0.83 ± 0.14
Proposed method	37.72 ± 3.1	0.91 ± 0.27

## Conclusion

In this work, we have proposed a new GAN-based SR method that outperforms other state-of-the-art super-resolution methods on different medical imaging modalities. We have used the concept of a multipath learning network to extract features at different scales and a progressive upscaling network to prevent the artifacts in the generated HR image. Furthermore, we have used L1 loss (mean absolute error) (mean absolute error) along with the the losses used in SRGAN, which generates more realistic and smooth HR images. We have evaluated the performance of the traditional SRGAN^31^, interpolation bases method (bicubic interpolation), and our proposed architecture on four publicly available medical imaging datasets. Experimental results on medical imaging datasets of different modalities have demonstrated the effectiveness of our proposed method. We have presented a fair comparison among all three methods, and from the results, the proposed architecture outperformed the other two methods on all the datasets. When transforming the LR image to an HR image, the proposed method can preserve fine details while improving the overall quality of the images. We believe that these results can be vital to advance the research on super-resolution of LR medical image data recorded in clinics.
